# Microbiota and clinical outcomes of implant‐supported full‐mandible dentures on patients with a history of periodontitis: A 5‐year prospective cohort study

**DOI:** 10.1111/jopr.14081

**Published:** 2025-06-06

**Authors:** Jefferson Antônio Gomes, Yumi Chokyu Del‐Rey, Alice Ramos de Freitas, Ivete Aparecida de Mattias Sartori, Cássio do Nascimento

**Affiliations:** ^1^ Department of Dental Materials and Prosthodontics Ribeirão Preto School of Dentistry, University of São Paulo Ribeirão Preto SP Brazil; ^2^ Implantology Area Latin American Institute of Research and Education in Dentistry Curitiba PR Brazil

**Keywords:** dental implants, microbiome, periodontitis, prosthodontics

## Abstract

**Purpose:**

To characterize the microbiota of implant‐supported full‐mandible dentures and its correlation to clinical outcomes for up to 5 years after implant loading in patients with a history of periodontal disease.

**Materials and Methods:**

Twelve individuals with a history of periodontitis (Stage IV and Grade B) received five immediate implants and a fixed complete‐arch mandibular prosthesis. Microbiological (total counts, microbial profile, and diversity) and clinical outcomes (probing depth, bleeding on probing, and bone resorption) were assessed before tooth extraction and at 4 and 5 years postloading. Thirty‐five microbial species were detected and quantified by DNA checkerboard analysis.

**Results:**

The microbiota significantly changed after 5 years of implant loading, with an increase in the abundance of periodontal pathogens and microbial diversity over time. The biofilm microbial profile and genome counts of implants were substantially different from the ones found in the remaining teeth at baseline, but no correlations between microbial taxa/counts and clinical outcomes were observed.

**Conclusion:**

The microbiota of dental implants was found to be different from the remaining teeth in patients with a history of periodontitis. Significant microbial shifts occurred in the microbiota of implants after 5 years of function; nonetheless, the microbiological changes did not impact clinical outcomes, which were consistent with health throughout the follow‐up period.

Implant‐supported restorations have been used worldwide to rehabilitate edentulous patients with predictable results. The estimated 5‐year implant survival rates (%) of removable and fixed implant‐supported prostheses in edentulous jaws were, respectively, 97.77% and 98.84%. If the maxilla and mandible were compared, the maxilla prostheses had a significantly higher implant loss rate. Concerning the implant numbers, the risk of implant failure is more than three times higher with restorations using less than four implants.^1^ Studies with longer follow‐up periods have also found very high rates reaching 100% in the fixed restorations.^2^ Studies reported cumulative survival rates of 98.6% after 6 years for fixed complete dental prostheses^3^ and 96.26% with a follow‐up period up to 9 years in partial extended fixed restorations.^4^ Nikellis et al. demonstrated minor prosthetic complications, high patient satisfaction, and a 100% survival rate throughout 12 years of follow‐up for complete fixed dentures.^5^

Although the predictability of dental implant survival and success is generally considered high, the literature indicates that a previous history of periodontitis may be an important risk factor for peri‐implantitis.^6^, ^7^ The main reason for this is that the microbiota that forms around dental implants is similar to the microbiota on the remaining compromised teeth.^8^ Once dental implants are placed, they are rapidly colonized by microorganisms, and a peri‐implant biofilm has been formed. Disruption of the oral microbiota balance followed by an increase in opportunistic pathogens may result in inflammatory reactions in the peri‐implant tissues and bone loss.^9^

Peri‐implantitis remains a therapeutic challenge for clinicians as a major cause of late dental implant failure;^10^ nonetheless, considerable advances have been achieved in the management of implant‐rehabilitated patients with a history of periodontal disease. A retrospective cohort study showed that the implant survival rate in these patients was 93.1% after 10 years, with no significant differences compared with nondiseased patients. The survival rates considering the prostheses extension were as follows: 97.7% for single‐unit, 95.3% for partial, and 97.8% for complete arch restorations.^11^ To date, however, the clinical outcomes of full‐arch rehabilitations on immediate implants in patients with a history of periodontitis remain unclear. Whether the microbiota of previously periodontally compromised sites undergoes relevant changes after implant placement and whether the acrylic material of dentures can influence the profile of the newly formed biofilm, remain to be elucidated.

It has been reported that the incidence of peri‐implantitis increases over time after functional loading in periodontally compromised patients.^12^ In this context, long‐term investigations of the correlation between microbiological and clinical findings over time may elucidate the relationships between microbial shifts, clinical outcomes, and prognosis of implant‐retained rehabilitation in these patients. The aim of this prospective clinical study was to characterize the microbiota of implant‐supported complete lower dentures from implant placement to 5 years after loading in patients with a history of periodontitis (Stage IV and Grade B) and to correlate the microbiological findings to clinical outcomes. The microbial diversity and potential microbial shifts over time were assessed by checkerboard DNA‐DNA hybridization. We hypothesized that oral microbiota in the immediate implants of complete lower dentures over time is similar to that of periodontitis affected‐dentition, with no changes in the disruption of the microbial composition.

## MATERIALS AND METHODS

### Participant recruitment and study plan

This prospective trial assessed the microbiota related to implant‐supported complete lower dentures of 12 participants (8 women and 4 men) diagnosed with periodontitis (Stage IV and Grade B) as defined by the American Academy of Periodontology.^13^ Partially edentulous patients were treated and rehabilitated at the Dental Clinic Latin American Institute of Research and Education in Dentistry (ILAPEO, Curitiba, Brazil) from July to November 2014; microbiological and clinical evaluations were performed from implant placement (baseline) to November 2019. At the time of implant placement, patients were aged between 46 and 76 years (mean age 60.14 ± 7.69 years) and met the following inclusion criteria: had a low to moderate rate of disease progression over time, generalized (> 30% of teeth affected) and severe clinical attachment loss (≥ 5 mm), probing pocket depth ≥ 6 mm, severe bone loss, clinical mobility, subgingival calculus, and bleeding on probing. Exclusion criteria were edentulous maxilla, pregnancy, lactation, periodontal or antibiotic therapy in the last 3 months, active infectious diseases, smoking, and inability to maintain good oral hygiene. The antagonist jaws (maxillae) presented complete natural teeth, or in some cases, a combination of natural teeth and single‐unit or short‐span fixed restorations. The treatment plan consisted of the extraction of compromised inferior teeth followed by immediate placement of implants and prosthetic loading. None of the participants presented contraindications to dental extractions and received proper local hygiene (site disinfection) to be better optimized before extraction was performed. Five implants were placed in the intraforaminal area. The full description of surgical and prosthetic procedures was previously published by Gomes et al.^14^ All surgical and prosthetic procedures were performed by the same clinician (J.A.G.). In brief, all participants received five immediate two‐piece morse taper implants (Titamax Cortical CM; Neodent, Brazil) in the interforaminal region at the alveolar bone level, followed by immediate loading with screw‐retained full‐arch mandible prostheses. Implant diameter and length ranged from 3.5 to 4.3 mm and 11 to 17 mm, respectively. Prostheses consisted of a cobalt‐chrome framework covered by thermoplastic acrylic resin.

The primary outcome, microbial count, was used for sample size estimation of this observational study. Sample size estimation (*N*) was based on the raw data from the previous study.^14^ The rank partial eta‐squared (rank‐based ANOVA) was used for the estimation of the effect size. Briefly, confidence intervals (95% CI: 0.88–1.00) and *p*‐values (two‐tailed) were computed using a Wald‐type distribution. Multilevel modeling (linear mixed models) was used considering the following parameters: number of clusters (time point and implant of sampling), predicted effect size of 1.69, intra‐class correlation of 0.1, two‐sided alternative hypothesis, significance level = 0.05, and power = 0.90. Sample size estimation resulted in 12 participants. The final sample size was increased by 30% (*N* = 16) to account for potential nonresponse or drop out. Sample size estimation was performed using R software (version 4.0.0; package lme4). The study was approved by the local ethics committee of the Faculty of Dentistry of Ribeirão Preto, under registration code CAAE 63508116.0.1001.5419. Informed and written consent was obtained from each participant, and the investigation was carried out in accordance with the Declaration of Helsinki. The study followed the STROBE statements for reporting observational studies.

### Follow‐up and data collection

Biofilm was collected from the remaining teeth immediately before extraction (T1), and from implants after 4 years (T2) and 5 years (T3) of implant loading. Clinical outcomes were also assessed at the same time‐points. All the biofilm sampling and clinical outcomes measurements were performed by the same clinician (J.A.G.). The participants received the same supportive hygiene care throughout the experimental period, which consisted of biofilm removal by polishing with a rubber cup and flossing, performed at 4 and 5 years before sampling.

For the microbiological analysis, subgingival biofilm samples from periodontal sulci of teeth (at baseline—T1) and from peri‐implantar sulci of 5 implants (at T2 and T3) were collected using #30 sterile paper points. Each tooth/implant sample was defined as a pool of 6 paper points placed into the sulcus for 30 s in the mesial, medial, and distal positions (3 in the buccal and 3 in the lingual aspects). A total of five sampling implants were investigated for each patient at the evaluated periods. After collection, samples were transferred to microtubes containing 150 µL of TE (10 mM Tris‐HCl, 1 mM EDTA, pH 7.6), followed by the addition of 150 µL of 0.5 M NaOH, and stored at −20°C until laboratory processing.

Patients were examined for the following clinical parameters: probing depth, bleeding on probing, and marginal bone loss. All measurements of probing depth and bleeding on probing were performed twice at the same six biofilm sampling sites by a calibrated clinician using a manual periodontal probe (Hu‐Friedy, USA). The peri‐implant marginal bone loss was evaluated by intraoral radiographs at the mesial and distal sites using the paralleling technique with an oral device that allowed standardization of film position throughout the experimental period. Marginal bone loss data were provided as the mean vertical and horizontal marginal bone loss measured using the software Image J Tool (Version 3.00 for Windows, University of Texas Health Sciences Center, USA).

### Microbiological analysis

The checkerboard DNA‐DNA hybridization method was used for identification and quantification of the microorganisms harboring the investigated microbiota. A set of 35 bacterial species, including periodontal pathogens and two *Candida* spp., were investigated: *Aggregatibacter actinomycetemcomitans, Bacteroides fragilis, Capnocytophaga gingivalis, Campylobacter rectus, Escherichia coli, Eikenella corrodens, Enterococcus faecalis, Fusobacterium nucleatum, Klebsiella pneumoniae, Lactobacillus casei, Mycoplasma salivarium, Pseudomonas aeruginosa, Pseudomonas putida, Peptostreptococcus anaerobius, Porphyromonas endodontalis, Porphyromonas gingivalis, Prevotella intermedia, Prevotella melaninogenica, Prevotella nigrescens, Parvimomas micra, Staphylococcus aureus, Strepetococcus gordonii, Strepetococcus mitis, Strepetococcus mutans, Strepetococcus oralis, Strepetococcus parasanguinis, Strepetococcus sanguinis, Strepetococcus salivarius, Strepetococcus sobrinus, Solobacterium moorei, Tannerella forsythia, Treponema denticola, Veillonella parvula, Candida albicans*, and *Candida dubliniensis*. Samples were processed as previously described.^15^ Briefly, DNA of clinical samples and mixtures of genomic DNA corresponding to either 10^5^ or 10^6^ microbial cells of each target species were hybridized against the set of 35 target probes overnight at 65°C. Positive hybridization signals were detected by exposing the membrane to Hyperfilm ECL (GE Healthcare, UK), and the number of microorganisms recovered from the clinical samples could be expressed as counts by comparing the chemiluminescence intensity signals of the test samples with the signals of the standard lanes. Microbial count analysis was performed using the software CLIQS—Core Laboratory Image Quantification Software (TotalLab Ltd, Newcastle upon Tyne, UK). Microbial diversity of the microbiota was first determined by measuring the relative abundances of microbial species. Shannon–Weaver and Simpson diversity indices were also determined to further infer the biofilms composition. The Jaccard coefficient was used to summarize the similarity of species occurrence in the different microbiota over time.

### Statistical analysis

Brunner‐Langer nonparametric analysis of longitudinal data in factorial experiments was used to determine the main and interaction effects of implants, target species, and time of functional loading on the microbial counts and clinical outcomes.^16^ Generalized estimating equations (GEE) modeling was performed for multiple comparisons to identify variables that were significantly associated with specific time points. Significance of diversity metrics was determined using ANOVA followed by Tukey's honest significant difference (HSD). Similarities of microbial profiles were assessed by the Jaccard coefficient. Correlations between microbiota and clinical outcomes were evaluated by Spearman`s rank correlation coefficient. Statistical significance was set at *p* < 0.05 level. All statistical analyses were performed using R statistical software with the *nparLD*, *ggpubr*, *geepack*, and *Vegan* packages (Version 4.0.0, R Foundation for Statistical Computing, Vienna, Austria).

## RESULTS

Of the initial 16 participants enrolled in the previous investigation, only 12 completed the 4‐ and 5‐year follow‐ups (mean age, 60.14; standard deviation, 7.69). Two individuals did not meet the criteria for microbiological sampling by 2014, and two could not be contacted in this current cohort study. A total of 180 biofilm sampling implants were investigated at the 3 sampling periods (5 teeth/implants × 12 individuals × 3 periods), yielding 6300 DNA checkerboard analyses (× 35 target species).

### Total microbial count

**FIGURE 1:**
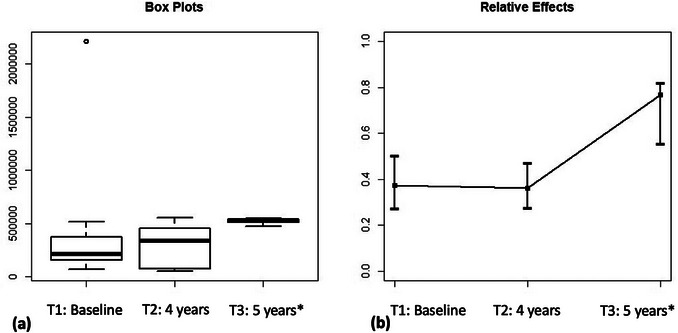
(a) Effect of time on total microbial count, expressed by medians, first and third quartiles, and minimum and maximum values measured at baseline (T1), after 4 years (T2) and after 5 years (T2), of implant function; (b) Pointwise 95% confidence intervals, indicating the lower limit, point estimate, and the upper limit for each period of investigation. *Significant differences detected by WTS/ATS followed by Bonferroni correction (T1 vs. T3: *p* = 1.22 × 10^−5^; T2 vs. T3: *p* = 1.24 × 10^−4^; T1 vs. T2: *p* = 0.690).

Data on the total microbial count consisted of a pool of all 35 target species investigated in this study. Figure 1 shows the relative effect of time on total microbial count along with the pointwise 95% confidence intervals without distinguishing between the 5 different implants. The box plots on the left panel (a) represent the median, first quartile, third quartile, minimum, and maximum distance measured at baseline (T1), and after 4 (T2) and 5 years (T2), respectively. They indicate that the measured distances increased over time, confirming the significance of the time effect. The dataset on the right panel (b) represents the 95% confidence intervals, indicating the lower limit, point estimate, and upper limit for each study period. The point estimates significantly increased at 5 years of investigation, indicating that the longer the evaluation period, the higher the observed microbial counts. Relative time effect significances were assessed by Wald‐Type Statistic (WTS) and ANOVA‐type statistic (ATS). Both yielded highly significant *p*‐values of 1.89 × 10^−6^ and 8.59 × 10^−7^, respectively. Multiple comparisons with Bonferroni correction showed that microbial count was higher at the 5‐year time point (T1 vs. T3: *p* = 1.22 × 10^−5^; T2 vs. T3: *p* = 1.24 × 10^−4^). No significant differences were found between the distributions of the measured counts for baseline and the 4‐year time point (*p* = 0.690).

**FIGURE 2:**
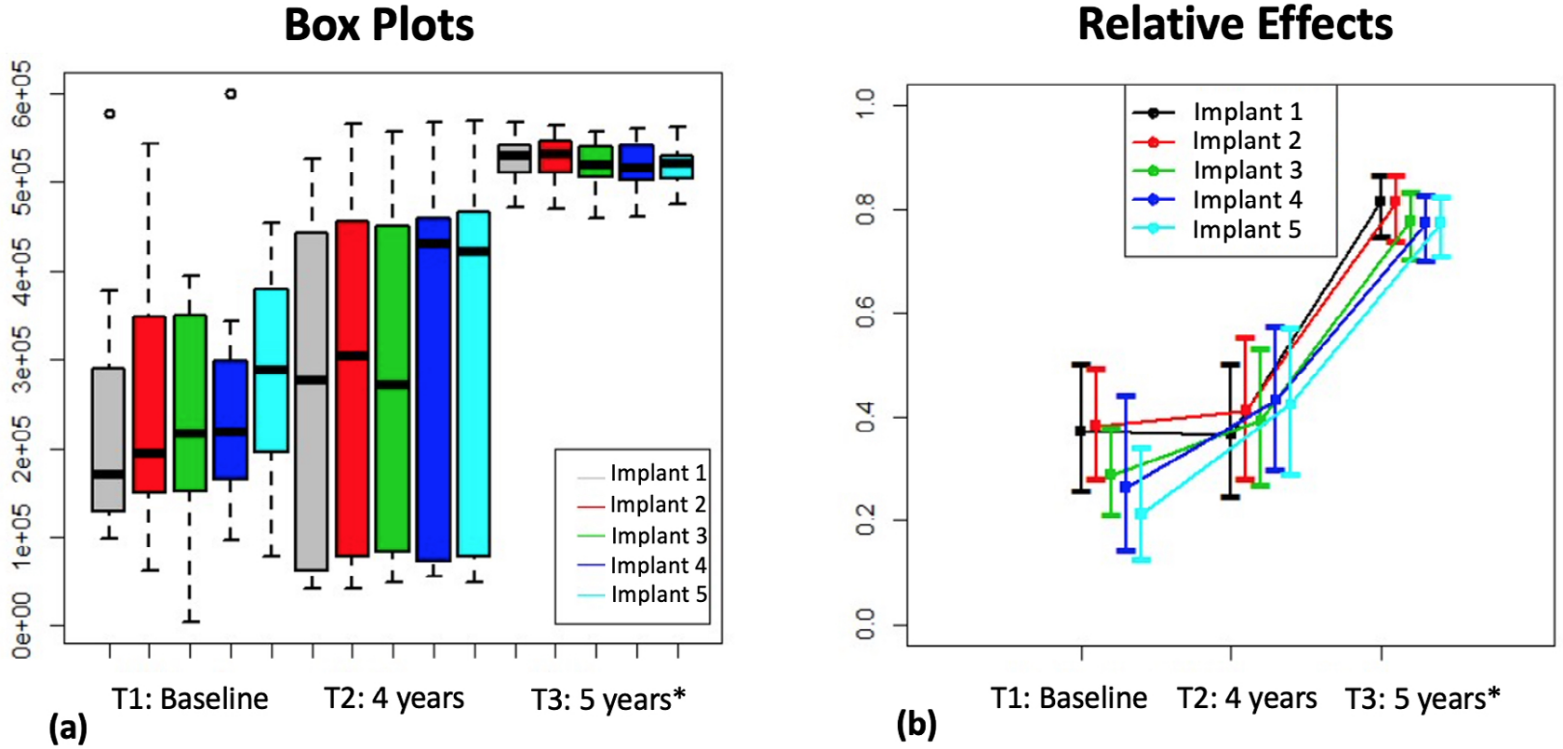
(a) Effect of sampling region on total microbial count at each time point; (b) 95% confidence intervals for total microbial count considering the different sampling implants and time points. *A significant time effect was detected by WTS (*p* = 4.06 × 10^−24^) and ATS (*p* = 4.06 × 10^−24^); T1 = T2 < T3.

Figure 2 shows the total microbial counts in the three study periods considering the five different sampling implants for each patient. Box plots and 95% confidence intervals for the relative effects of the implants at each time point are displayed in the left (a) and right panels (b), respectively. No significant relative effect was observed for implants (WTS: *p* = 0.9815; ATS: 0.9787). Also, no statistical interaction was found between the implant and time variables (WTS: *p* = 0.8209; ATS: *p* = 0.8531), indicated by the quasi‐parallel time profiles on the right panel (b). Conversely, a highly significant time effect was confirmed by WTS (*p* = 4.06 × 10^−24^) and ATS (*p* = 4.06 × 10^−24^) with higher microbial counts recorded at the 5‐year time point. The 95% confidence intervals further confirm that the total microbial counts were shown to increase at the 5‐year follow‐up regardless of the implant location.

### Individual microbial counts

**FIGURE 3:**
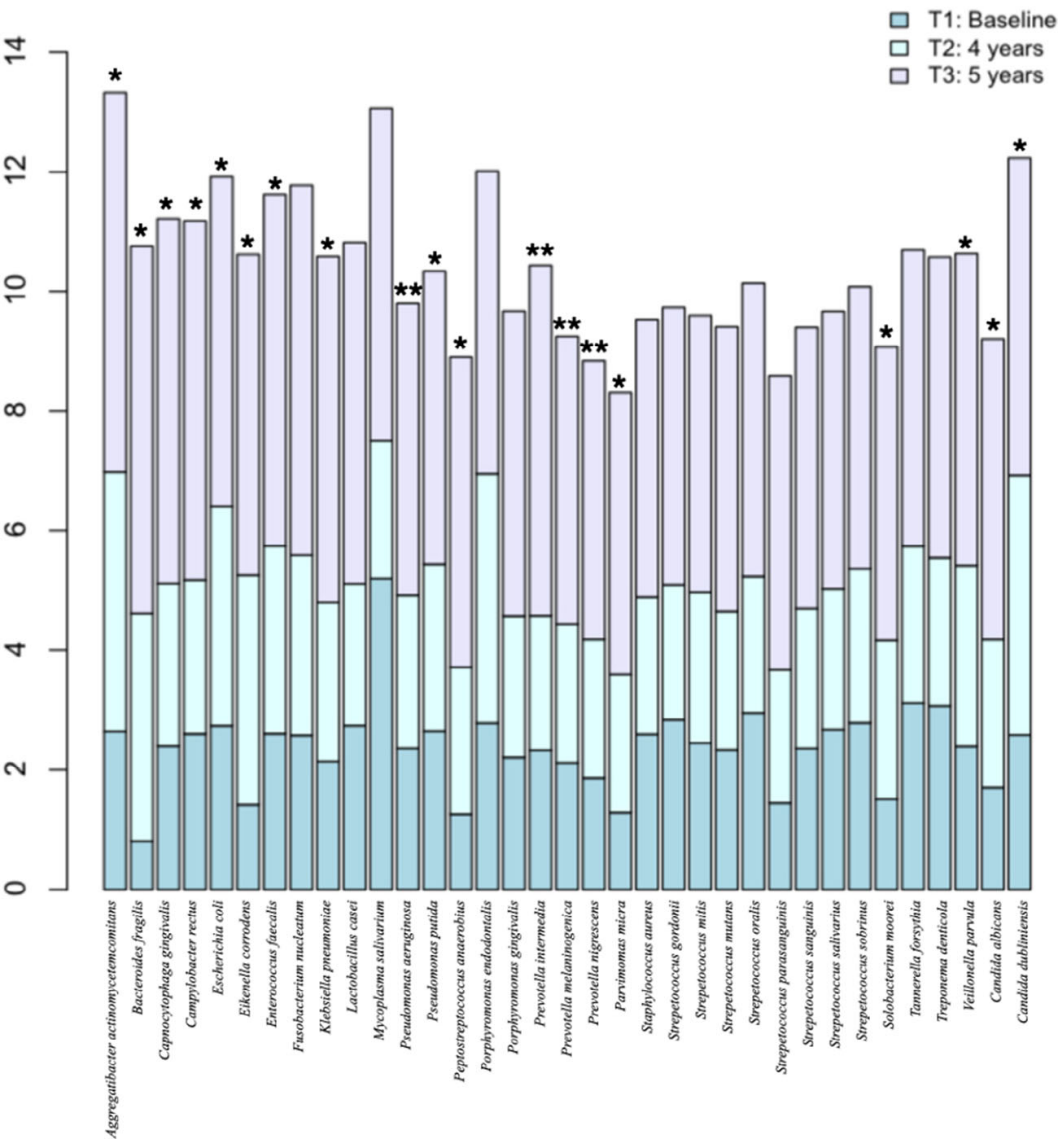
Individual microbial counts (median, ×105) of the 35 target species detected on subgingival (T1) and peri‐implant biofilms (T2; T3) over time. Significant differences were verified by generalized estimating equations; **p < 0.01; *p < 0.0001.

The modified ATS for whole‐plot factors showed a significant relative effect for the species variable. Both WTS and ATS indicated a strong interaction between species and time at the 5% level, with *p*‐values of 5.92 × 10^−323^ and 2.16 × 10^−69^, respectively. Moreover, significant time (*p* = 2.00 × 10^−16^) and species (*p* = 0.0001) effects were observed; therefore, the significance of these relative effects was further investigated. GEE modeling was applied to evaluate the effects while accounting for the longitudinal pattern of data and clustering within subjects. Nineteen of the 35 microbial species investigated were found to increase in number at the 5‐year time point. The individual microbial counts and significance for each target species are shown in Figure 3. No significant differences were found comparing species between baseline and after 4 years of loading (*p* > 0.05). The most common periodontal pathogens were found in elevated levels (median, ×10^5^ cells) at the 5‐year time point; *P. gingivalis* (4.89; *p* = 2.11 × 10^−6^), *T. forsythia* (4.90; *p* = 5.30 × 10^−11^), and *T. denticola* (4.97; *p* = 0.0035). Other relevant bacterial species commonly related to peri‐implant diseases were also found in high levels at the 5‐year time point; *P. intermedia* (5.10; *p* = 0.0246), *F. nucleatum* (6.20; *p* < 2.00 × 10^−16^), and *A. actinomycetemcomitans* (6.34; *p* = 2.84 × 10^−12^). The median, minimum, and maximum values (×10^5^ cells) of microbial species at baseline and 4‐year follow‐up ranged from 2.74 (minimum: 0; maximum: 15.77) to 3.93 (minimum: 0; maximum: 6.67).

### Diversity and similarity of microbial communities

**FIGURE 4:**
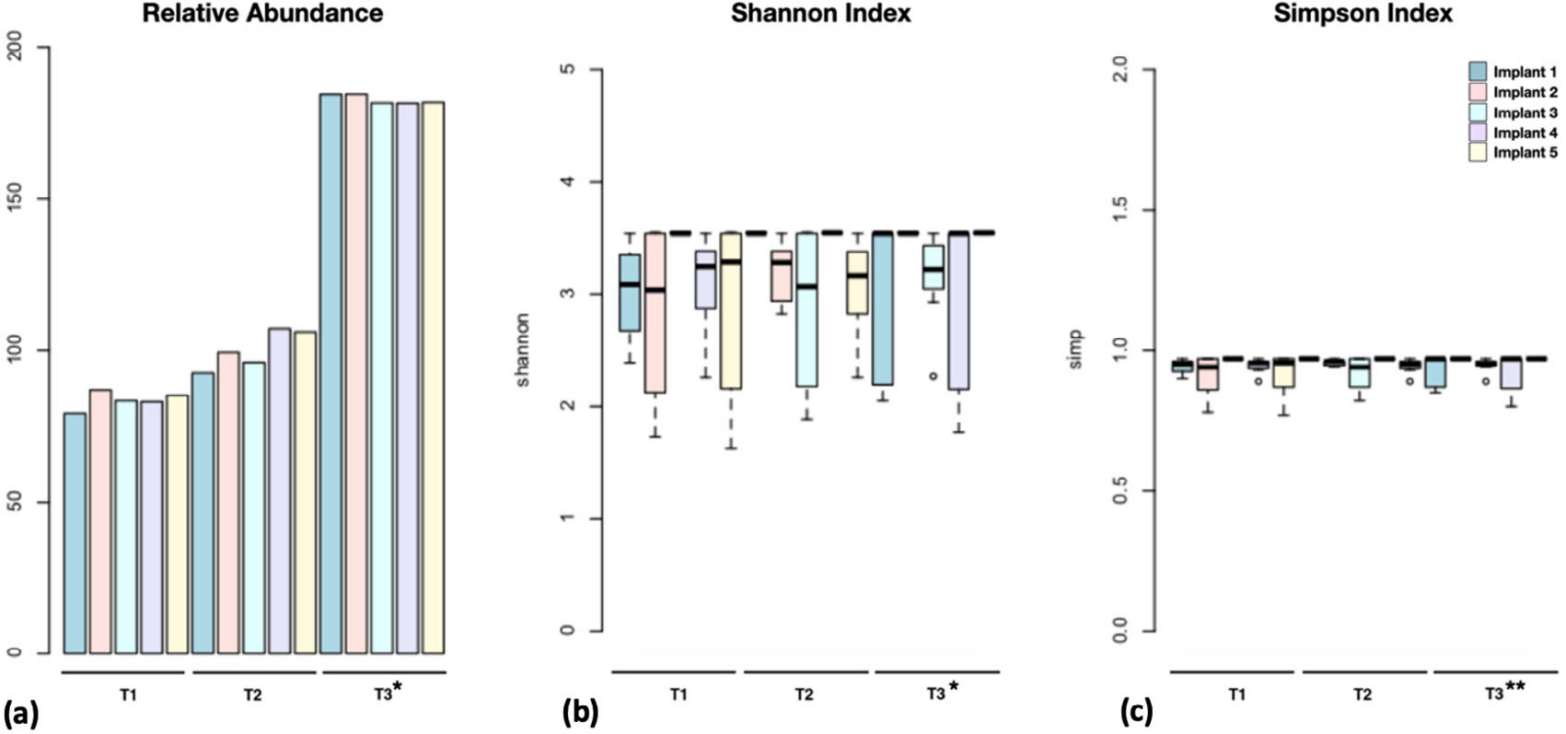
Diversity of the investigated biofilms. (a) Proportional abundance of the total microbial community within each sample; (b) Shannon; and (c) Simpson alpha diversity indices. Differences were detected by ANOVA followed by Tukey's HSD; **p < 0.01; *p < 0.0001.

The diversity of the investigated biofilms was represented by the proportional abundance of the total microbial community within each sample and by Shannon and Simpson alpha indices (within‐sample diversity). All diversity data are shown in Figure 4. The proportion of the total microbial community was highest in samples from the 5‐year time point, regardless of implants (*p* = 1.32 × 10^−11^). All target species had moderate to high abundance values. The highest relative abundances (median relative abundance > 70%) at the 5‐year time point were recorded for *A. actinomycetemcomitans*, *F. nucleatum*, and *P. melaninogenica*; overall, the median relative abundance of species at the 5‐years follow‐up was 61.4%. The abundance of samples from the 4‐year time point was higher than baseline, but differences were not significant (*p* > 0.05). Consistent with the abundance results, ANOVA followed by Tukey's HSD confirmed that alpha diversity, as measured by Shannon and Simpson indices, was higher after 5 years (*p* = 3.60 × 10^−11^ and *p* = 0.0152 for Shannon and Simpson, respectively). The Shannon index ranged from 3.47 to 3.55, indicating high alpha diversity in all samples, regardless of sampling time. Corroborating these results, the Simpson index also presented high values, ranging from 0.96 to 0.97, with a mean of 0.96. Results suggest that there were no dominant species in the microbiota of participants in any of the periods evaluated.

**FIGURE 5:**
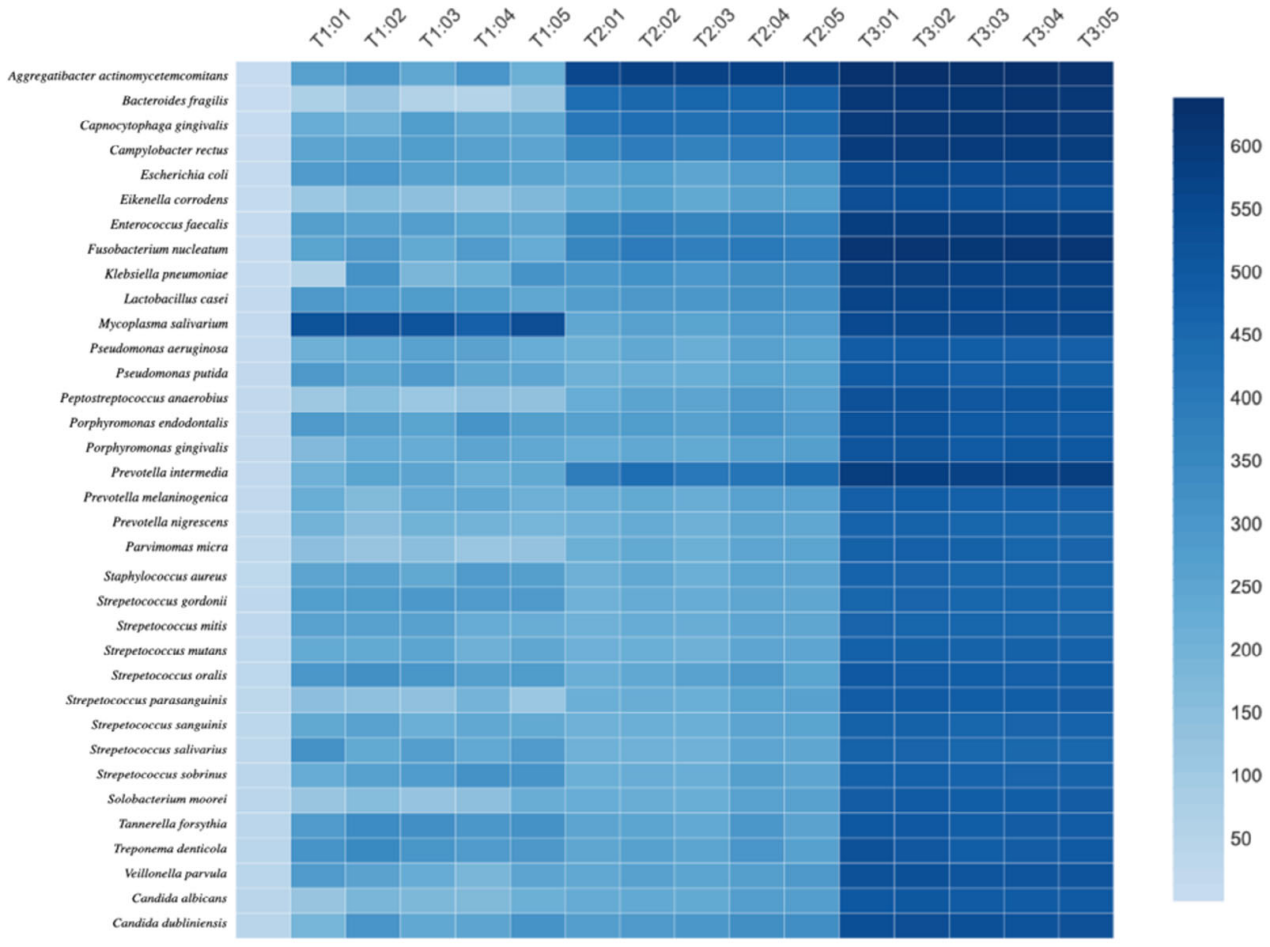
Heatmap representation of microbiota similarity between implants over time based on a community structure analysis at the species level using Jaccard metrics (T1: Baseline, T2: 4 years, and T3: 5 years of implant loading; 01 to 05: implants).

The number of shared species and their relative abundances were also measured using the Jaccard similarity coefficient. Based on the Jaccard metrics, distinct patterns of similarity and dissimilarity were found, clustering samples from the different time points (Figure 5). Significant differences in community affiliation and structure were detected over time. The microbiota found after 5 years of implant loading did not show much similarity to either of the two previous time points. In general, pairwise similarities were recorded as follows: T1‐T2 (63%), T1–T3 (43%), and T2–T3 (53%). Similarity decreased over time; after 5 years of function (T3), the microbiota of dental implants shared less than half of the members present in teeth at the baseline (T1). Early colonizers belonging to the genera *Streptococcus* spp. and late colonizers represented mainly by gram‐negative genera (including periodontal pathogens like *P. gingivalis*, *T. forsythia*, *T. denticola*, and *A. actynomycetemcomitans*) were both found in higher abundance at the 5‐year follow‐up. No significant differences were observed within implants, which showed high similarity (between 90% and 99%).

### Clinical outcomes and correlation with microbiological findings

**FIGURE 6:**
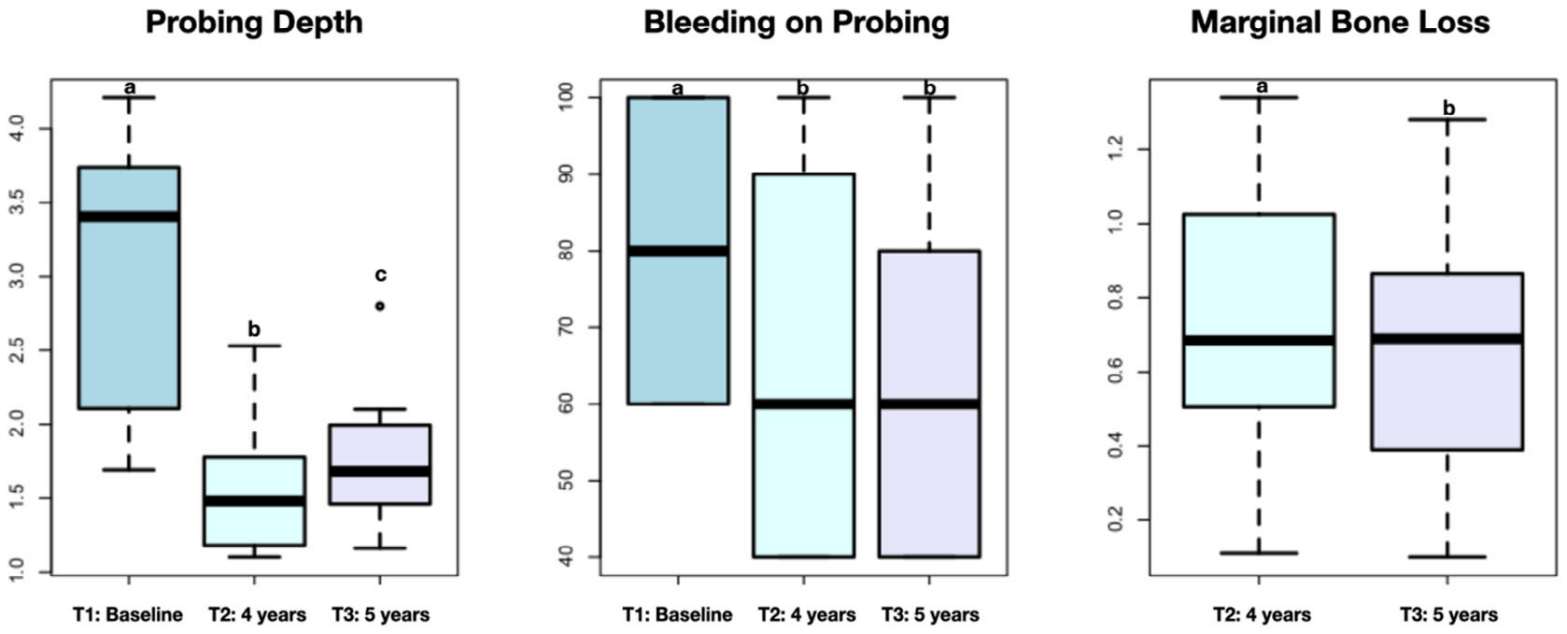
Medians, maximum and minimum values, upper and lower quartiles of probing depth, bleeding on probing, and marginal bone loss over time. Different letters mean significant differences detected by WTS/ATS: a > c > b; *p* < 0.05.

No clinical complications occurred in any patient or implant throughout the entire 5‐year follow‐up. No signs of local bacterial infection, mucositis, or peri‐implantitis were detected. Overall, personal hygiene care was considered satisfactory in all participants. The median, first quartile, third quartile, minimum, and maximum values of probing depth (mm), bleeding on probing (%), and marginal bone loss (mm) of teeth before extraction and implants are shown in Figure 6. Probing depth of teeth at baseline and implants after 5 years indicated periodontal health (mean values of 3.04 ± 0.92 mm for teeth and 1.74 ± 0.45 mm for implants). The mean percent of bleeding on probing was 81.66% for teeth at baseline and 63.33% for implants at 5‐year follow‐up. The mean total marginal bone loss (mm, ± SD) around dental implants after 5 years was 1.39 ± 0.73. The *p*‐values of pairwise comparisons were probing depth: T1–T2: *p* = 1.30 × 10^−6^; T1–T3: *p* = 0.0006; T2–T3: *p* = 1.27 × 10^−8^; bleeding on probing: T1–T2: *p* = 0.0005; T1–T3: *p* = 0.0111; T2–T3: *p* = 0.89; marginal bone loss: T2–T3: *p* = 0.038).

Spearman's rank correlation test was performed to analyze the association between total microbial count and clinical outcomes after 5 years. None of the evaluated clinical parameters were significantly correlated with the installed microbiota: probing depth (*R* = 0.26; *p* = 0.23), bleeding on probing (*R* = −0.31; *p* = 0.14), and marginal bone loss (*R* = 0.28; *p* = 0.21).

## DISCUSSION

Immediate implant placement with immediate loading has been considered a treatment option for the rehabilitation of periodontally affected individuals. Most studies in the literature report high survival rates for one‐step rehabilitation approaches in these patients and confirm that implants are successfully osseointegrated and maintained in the oral cavity.^17^, ^18^ Nonetheless, there is still a lack of studies with longer follow‐up periods that assess the microbial shifts over time of implant function and the impact of bacterial communities composition on clinical outcomes. Dysbiosis of the oral microbiota, commonly characterized by a loss of microbial diversity with the overgrowth and overrepresentation of pathogenic species, has been considered the main etiological factor for peri‐implantitis.^19^ Although the mechanism by which dysbiosis occurs in peri‐implantitis is still not clear, understanding the dynamic interactions between the installed microbiota and host tissues over time is crucial to reduce risks related to potential microbial imbalances and to develop predictive models for diagnosis and treatments. Thus, the aim of this study was to prospectively investigate the oral microbiota formed around dental implants supporting full‐mandible restorations in individuals with a history of periodontitis and determine whether it was correlated with the clinical outcomes after 5 years of functional loading.

The hypothesis of this study was confirmed since the results demonstrated that the microbiota of teeth at baseline significantly changed after 5 years of implant placement, and shifts in bacterial community composition were not associated with peri‐implant diseases. Microbial counts and communities around dental implants remained similar to those at baseline until the 4‐year time point and then increased significantly after 5 years. The closely related periodontal pathogens *T. denticola*, *F. nucleatum*, *P. gingivitis*, and *A*. *actinomycetemcomitans* were found in high levels and abundance, suggesting that the history of periodontitis has a relevant impact on the late peri‐implant microbiota and that microbial levels increase the longer implants are in function, as previously reported.^20^, ^21^ Indeed, these alterations might be involved in the progression of peri‐implant diseases and may represent a potential risk for the long‐term success of dental implants.^22^ Interestingly, the microbial diversity also increased over time, reaching the high values at the 5‐year time point, which may inhibit the outgrowth of pathogenic microorganisms while favoring the maintenance of microbiota balance. This pattern may be a rationale for the nonsymmetric variability of microbial data over time. Skewed distributions of interquartile range and whiskers were observed in the different time points. At baseline, the box plot shape presents a positive skew, while at 4‐year follow‐up it presents a negative skew. For both the baseline and 4‐year time point, the data distribution is stretched. Conversely, at the 5‐year time point, the distribution is quite squeezed. Literature has shown that more diverse microbial communities tend to be more stable in their composition over time.^23^

In this study, the installed microbiota did not promote substantial clinical changes over time. All participants presented no sign of infection/inflammation, and data on clinical parameters were compatible with healthy conditions during the entire experimental period. Despite the differences comparing both probing depth and bleeding on probing at baseline and 4‐year and 5‐year time points, we should consider that measurements were taken from teeth at baseline. Compared to data from the previous clinical trial study, both probing depth and bleeding on probing of implants were quite similar in all the follow‐up periods (4/8 months and 4/5 years). No significant correlation was found between mucositis or peri‐implantitis and the microbiota profile. Noteworthy, these findings confirm that the mere presence of pathogens in the peri‐implant biofilm is not necessarily associated with disease development and implant failure.^24^, ^25^

The authors have previously demonstrated that the peri‐implant microbiota of individuals with a history of periodontitis at early stages of functional loading (up to 8 months) was composed mainly of gram‐positive bacteria belonging to the *Streptococcus* genus and by gram‐negative genera, such as *Veillonella*, *Porphyromonas*, and *Prevotella*; some periodontopathogenic species (i.e., *T. denticola*, *F. nucleatum*, *P. gingivitis*, and *A*. *actinomycetemcomitans*) were also observed in low to moderate levels.^13^ From that short period of investigation, it was concluded that the microbiota of dental implants was quite similar to that found in the extracted teeth before implant placement, being characterized by healthy microbial patterns. Studies have demonstrated that commensal (or normal microflora) and pathogenic species may be present in moderate to high levels even in early biofilm and healthy conditions.^26^, ^27^ Conversely, our current findings suggest that both the microbiota of the remaining teeth and the implant loading time may have a relevant impact on the peri‐implant microbiota of individuals with a history of periodontal diseases. Specifically, the time factor may determine the extent to which the microbiota of the remaining teeth may influence the long‐term biofilm formation. Therefore, it seems to be extremely relevant to prevent an imbalance of the oral microbiota by modulating the growth of pathogenic bacteria with specific interventions, such as biofilm control. The presence of deep sulci favors the proliferation of anaerobic species, and the literature highlights the importance of supportive hygiene protocols for the maintenance of implants for individuals with a history of periodontal diseases.^21^ However, a standardized protocol is still not available based on the current literature evidence.^28^ According to previous studies, a deeper peri‐implant sulcus is correlated to microbial shifts and increased levels of dysbiosis.^19^ In this study, a supportive hygiene protocol was performed during the entire experimental period, professional cleaning during the sampling, and participants were also instructed to maintain good personal oral hygiene. According to literature, personal hygiene plays an important role in health maintenance. This approach may explain the high microbial diversity and the microbiota balance observed over time. Regardless of the elevated levels of pathogenic species after 5 years of loading, the high abundance of diverse commensal bacteria inhibited the proliferation of pathogenic species, preventing the development of disease. Commensal and pathogenic microorganisms are commonly found in the oral cavity in a symbiotic relationship, with the commensal species acting as competitors for the adhesion of more pathogenic microorganisms to mucosal and nonshedding surfaces.^29^ At the end of the experimental period, the mean probing depth around implants was lower than 1.8 mm, indicating peri‐implant health.

Relevant insights into the pathogenesis of peri‐implantitis can be drawn from this study; nonetheless, this investigation presented some limitations. The periodontal status and the biofilm on the maxillary remaining teeth were not investigated. Patients received conventional therapy at the beginning of the investigation, which consisted of supragingival cleaning and subgingival curettage. Sampling of the antagonists over time could help elucidate whether the microbiota of teeth can change over time and whether it can influence the peri‐implant microbiota. According to the literature, the microbiota of remaining teeth acts as a source of pathogenic species and may directly influence the microbiota of implants.^30^ The findings of this study are possibly a result of the translocation of microorganisms from the subgingival biofilm of the upper teeth to the peri‐implant sulci. Furthermore, the effect of interpersonal variation on the microbial levels and microbiota diversity was not investigated. Data on these aspects may be relevant, since interpersonal variation has been previously shown to influence the overall microbiota composition.^31^ Another limitation is related to the molecular method used to identify and quantify the target species. Checkerboard DNA‐DNA hybridization has a detection threshold of > 10^4^ cells, and cross‐reactions may occur among closely related taxa. In this study, the sensitivity was adjusted to detect DNA amounts corresponding to 10^5^ and 10^6^ cells, while maintaining enough specificity to discriminate between different genera. The hybridization signals equivalent to 10^4^ cells did not result in detectable or reproducible signals. However, the method does have advantages. DNA probes allow the simultaneous analysis of the microbial profile of up to 28 clinical samples for a wide range of bacterial species (up to 45 at the same time). In addition, species abundance, diversity, and communities may be computed from data recovered using checkerboard DNA‐DNA hybridization. Finally, due to the nature of the study, a continuation of a previous clinical trial, the number of patients enrolled may be considered a certain limitation. However, the sample size sufficiency met the research design requirements and did not compromise the quality of the analyses. The effect size resulting from the current study yielded 1.77, resulting in a stronger post hoc power of 0.98.

The results of this 5‐year follow‐up study suggest that the reversion of the microbiota to its predisease conditions may occur slowly and may not have a significant effect on clinical outcomes, probably due to the hygiene maintenance protocol. The results also indicate the relevance of hygiene therapies and their beneficial clinical impact through direct effects on biofilm control. Further studies with longer evaluation periods should extend these findings by investigating whether other potential microbial shifts may have an impact on the clinical outcomes.

## CONCLUSION

A significant microbial shift occurred in the bacterial communities of dental implants over time and resulted in increased bacterial abundance and a high alpha diversity index. Nevertheless, the microbial changes did not result in microbial dysbiosis and had no influence on clinical outcomes, which were compatible with healthy conditions throughout the 5‐year experimental period. In this context, these results suggest that immediate implant‐supported full‐arch restorations represent a predictable treatment in individuals with a history of periodontitis who were in a maintenance hygiene program.

## CONFLICT OF INTEREST STATEMENT

The authors declare no conflicts of interest.

## References

[1] Kern JS , Kern T , Wolfart S , Heussen N . A systematic review and meta‐analysis of removable and fixed implant‐supported prostheses in edentulous jaws: post‐loading implant loss. Clin Oral Implants Res. 2016;27: 174–195 25664612 10.1111/clr.12531 PMC5024059

[2] Alshiddi IF . Survival rate and clinico‐radiographic parameters around narrow‐diameter dental implants for fixed dental prostheses in the posterior regions: a systematic review. Dent Med Probl. 2023;60:345–353 37669472 10.17219/dmp/140757

[3] Papaspyridakos P , Sinada N , Ntovas P , Barmak AB , Chochlidakis K . Zirconia full‐arch implant prostheses: survival, complications, and prosthetic space dimensions with 115 edentulous jaws. J Prosthodont. 2025;34:271–280 39136214 10.1111/jopr.13922

[4] Chochlidakis K , Fraser D , Lampraki E , Einarsdottir ER , Barmak AB , Papaspyridakos P , et al. Prosthesis survival rates and prosthetic complications of implant‐supported fixed dental prostheses in partially edentulous patients. J Prosthodont. 2020;29:479–488 32364656 10.1111/jopr.13185

[5] Nikellis T , Lampraki E , Romeo D , Tsigarida A , Barmak AB , Malamou C , et al. Survival rates, patient satisfaction, and prosthetic complications of implant fixed complete dental prostheses: a 12‐month prospective study. J Prosthodont. 2023;32:214–220 35964246 10.1111/jopr.13593

[6] de Araújo Nobre M , Mano Azul A , Rocha E , Maló P . Risk factors of peri‐implant pathology. Eur J Oral Sci. 2015;123:131–139 25894059 10.1111/eos.12185

[7] Monje A , Aranda L , Diaz KT , Alárcon MA , Bagramian RA , Wang HL , et al. Impact of maintenance therapy for the prevention of peri‐implant diseases: a systematic review and meta‐analysis. J Dent Res. 2016;95: 372–379 26701350 10.1177/0022034515622432

[8] Mombelli A , Décaillet F . The characteristics of biofilms in peri‐implant disease. J Clin Periodontol. 2011;38:203–213 21323716 10.1111/j.1600-051X.2010.01666.x

[9] Nícoli LG , Malzoni CMA , Costa Neto PFD , Marcantonio C , Pigossi SC , Rösing CK , et al. Patient‐, implant‐ and prosthetic‐related factors on peri‐implant mucositis and bone loss. Braz Oral Res. 2024;38:e040 38747827 10.1590/1807-3107bor-2024.vol38.0040 PMC11376655

[10] Derks J , Tomasi C . Peri‐implant health and disease. A systematic review of current epidemiology. J Clin Periodontol. 2015;42:S158–S171 25495683 10.1111/jcpe.12334

[11] Correia F , Gouveia S , Felino AC , Costa AL , Almeida RF . Survival rate of dental implants in patients with history of periodontal disease: a Retrospective Cohort Study. Int J Oral Maxillofac Implants. 2017;32:927–934 28708925 10.11607/jomi.3732

[12] Pandolfi A , Rinaldo F , Pasqualotto D , Sorrentino F , La Torre G , Guerra F . A retrospective cohort study on peri‐implant complications in implants up to 10 years of functional loading in periodontally compromised patients. J Periodontol. 2019;91:995–1002. 10.1002/JPER.18-0715 31860130

[13] Caton JG , Armitage G , Berglundh T , Chapple ILC , Jepsen S , Kornman KS , et al. A new classification scheme for periodontal and peri‐implant diseases and conditions—introduction and key changes from the 1999 classification. J Clin Periodontol. 2018;45(Suppl 20):S1–S8 29926489 10.1111/jcpe.12935

[14] Gomes JA , Sartori IAM , Able FB , de Oliveira Silva TS, do Nascimento C. Microbiological and clinical outcomes of fixed complete‐arch mandibular prostheses supported by immediate implants in individuals with history of chronic periodontitis. Clin Oral Implants Res. 2017;28:734–741 27167329 10.1111/clr.12871

[15] do Nascimento C , de Albuquerque RF Jr , Monesi N , Candido‐Silva JA . Alternative method for direct DNA probe labeling and detection using the checkerboard hybridization format. J Clin Microbiol. 2010;48:3039–3040 20554808 10.1128/JCM.00390-10 PMC2916595

[16] Brunner E , Domhof S , Langer F . Nonparametric analysis of longitudinal data in factorial experiments. New York: Wiley; 2012.

[17] Chrcanovic BR , Martins MD , Wennerberg A . Immediate placement of implants into infected sites: a systematic review. Clin Implant Dent Relat Res. 2015;17:e1–e16 23815434 10.1111/cid.12098

[18] Sarafidou K , Lazaridi I , Gotsis S , Kirmanidou Y , Vasilaki D , Hirayama H , et al. Tooth preservation vs. extraction and implant placement in periodontally compromised patients: a systematic review and analysis of studies. J Prosthodont. 2022;31:e87–e99 35794083 10.1111/jopr.13560

[19] Kröger A , Hülsmann C , Fickl S , Spinell T , Huttig F , Kalfmann F , et al. The severity of human peri‐implantitis lesions correlates with the level of submucosal microbial dysbiosis. J Clin Periodontol. 2018;45:1498–1509 30341964 10.1111/jcpe.13023

[20] Lee KH , Maiden MF , Tanner AC , Weber HP . Microbiota of successful osseointegrated dental implants. J Periodontol. 1999;70:131–138 10102550 10.1902/jop.1999.70.2.131

[21] Smith MM , Knight ET , Al‐Harthi L , Leichter JW . Chronic periodontitis and implant dentistry. Periodontol 2000. 2017;74:63–73 28429486 10.1111/prd.12190

[22] Renvert S , Quirynen M . Risk indicators for peri‐implantitis. A narrative review. Clin Oral Implants Res. 2015;26:15–44 10.1111/clr.12636 26385619

[23] Flores GE , Caporaso JG , Henley JB , Rideout JR , Domogala D , Chase J , et al. Temproal variability is a personalized feature of the human microbiome. Genome Biol. 2014;15:531 25517225 10.1186/s13059-014-0531-y PMC4252997

[24] Sbordone L , Barone A , Ciaglia RN , Ramaglia L , Iacono VJ . Longitudinal study of dental implants in a periodontally compromised population. J Periodontol. 1999;70:1322–1329 10588495 10.1902/jop.1999.70.11.1322

[25] Graetz C , El‐Sayed KF , Geiken A , Plaumann A , Salzer S , Behrens E , et al.. Effect of periodontitis history on implant success: a long‐term evaluation during supportive periodontal therapy in a university setting. Clin Oral Investig. 2018;22:235–244 10.1007/s00784-017-2104-4 28353021

[26] Nascimento CD , Pita MS , Santos Ede S , et al. Microbiome of titanium and zirconia dental implants abutments. Dent Mater. 2016;32:93–101 26616687 10.1016/j.dental.2015.10.014

[27] de Oliveira Silva TS , de Freitas AR , de Albuquerque RF , Pedrazzi V , Ribeiro RF , do Nascimento C . A 3‐year longitudinal prospective study assessing microbial profile and clinical outcomes of single‐unit cement‐retained implant restorations: zirconia versus titanium abutments. Clin Implant Dent Relat Res. 2020;22:301–310 32026617 10.1111/cid.12888

[28] Soares PM , Silveira GDA , Gonçalves LS , Bacchi A , Pereira GKR . Maintenance protocols for implant‐supported dental prostheses: a scoping review. J Prosthet Dent. 2024;132:59–71 36535881 10.1016/j.prosdent.2022.08.026

[29] Avila M , Ojcius DM , Yilmaz O . The oral microbiota: living with a permanent guest. DNA Cell Biol. 2009;28:405–411 19485767 10.1089/dna.2009.0874 PMC2768665

[30] Ong CT , Ivanovski S , Needleman IG , Retzepi M , Moles DR , Tonetti MS , et al. Systematic review of implant outcomes in treated periodontitis subjects. J Clin Periodontol. 2008;35:438–462 18433385 10.1111/j.1600-051X.2008.01207.x

[31] Yu XL , Chan Y , Zhuang L , Lai HC , Lang NP , Leung WK , et al. Intra‐oral single‐site comparisons of periodontal and peri‐implant microbiota in health and disease. Clin Oral Implants Res. 2019;30:760–776 31102416 10.1111/clr.13459

